# Timing of surgery for hip fracture and in-hospital mortality: a retrospective population-based cohort study in the Spanish National Health System

**DOI:** 10.1186/1472-6963-12-15

**Published:** 2012-01-18

**Authors:** Julián Librero, Salvador Peiró, Edith Leutscher, Juan Merlo, Enrique Bernal-Delgado, Manuel Ridao, Natalia Martínez-Lizaga, Gabriel Sanfélix-Gimeno

**Affiliations:** 1 Centro Superior de Investigación en Salud Pública (CSISP), Valencia, Spain; 2 Instituto de Investigación Sanitaria Aragón, Zaragoza, Spain; 3 Grupo de Variaciones en la Práctica Médica en el Sistema Nacional de Salud; 4 Unit for Social Epidemiology, Faculty of Medicine, Lund University, Malmö, Sweden

## Abstract

**Background:**

While the benefits or otherwise of early hip fracture repair is a long-running controversy with studies showing contradictory results, this practice is being adopted as a quality indicator in several health care organizations. The aim of this study is to analyze the association between early hip fracture repair and in-hospital mortality in elderly people attending public hospitals in the Spanish National Health System and, additionally, to explore factors associated with the decision to perform early hip fracture repair.

**Methods:**

A cohort of 56,500 patients of 60-years-old and over, hospitalized for hip fracture during the period 2002 to 2005 in all the public hospitals in 8 Spanish regions, were followed up using administrative databases to identify the time to surgical repair and in-hospital mortality. We used a multivariate logistic regression model to analyze the relationship between the timing of surgery (< 2 days from admission) and in-hospital mortality, controlling for several confounding factors.

**Results:**

Early surgery was performed on 25% of the patients. In the unadjusted analysis early surgery showed an absolute difference in risk of mortality of 0.57 (from 4.42% to 3.85%). However, patients undergoing delayed surgery were older and had higher comorbidity and severity of illness. Timeliness for surgery was not found to be related to in-hospital mortality once confounding factors such as age, sex, chronic comorbidities as well as the severity of illness were controlled for in the multivariate analysis.

**Conclusions:**

Older age, male gender, higher chronic comorbidity and higher severity measured by the Risk Mortality Index were associated with higher mortality, but the time to surgery was not.

## Background

The incidence of hip fracture in Spain is higher than 100 per 100,000 inhabitants-year, exceeding 500 per 100,000 in people aged 65 and over [1,2]. The ratio men/women ranges between 2.5 and 3 [1,2], the variation among geographical areas being smaller than for other conditions [3]. Mortality in the month following the fracture ranges from 5% to 10%, reaching 30% after a year [4-6], with another 30% of patients having a high grade of disability [4,5,7]. In-hospital mortality varies greatly between series, ranging from 3.7% [8] to 12% [9].

The non-surgical repair of hip fracture is uncommon because of unacceptable outcomes [10]. A decision on the surgical modality depends on the fracture itself and patient factors like age. Whereas reduction and external fixation is a common option for young people or in trochanteric fractures, hip arthroplasty is usually a better option in elderly people with intracapsular fractures due to frequently associated necrosis. Fluid reposition, tromboembolic and antibiotic prophylaxis and early mobilization are accepted complementary treatments needed to get better outcomes [10].

The timeliness of surgery has been suggested as a critical aspect in the prognosis of hip fracture repair. In fact, several guidelines recommend performing surgery as soon as possible, preferably in the first 24 hours, suggesting that early surgery is associated with fewer complications, lower mortality and a reduced length of stay [11-13]. Early surgery has also been included as a quality marker in the highly disseminated set of Inpatient Quality Indicators from the Agency for Healthcare Research and Quality [14]. Nevertheless, whether early surgery is beneficial or not is a long-running controversy, and studies examining the relationship between surgery timing and outcomes show contradictory results. Combining studies selected in five literature reviews [15-19] with a search for the most recently published studies [20-26], we found 59 papers (with very different designs and methodological quality) analyzing the relationship between early surgery and mortality. Twenty-five studies found that early surgery was associated with a significant reduction in mortality, whereas 32 did not show such a protective association, and 2 of them even found a statistically significant higher mortality associated with early surgery. Despite this contradictory evidence, early repair has been adopted as a quality indicator in several of the Spanish Regional Health Services, occasionally being incorporated as a basis for pay for performance schemes for orthopaedic surgeons.

The aim of this study is to analyze the association between early hip fracture repair and in-hospital mortality in elderly people attending in public hospitals in the Spanish National Health System (sNHS) and, secondly, we aim to explore factors associated with the decision to perform early hip fracture repair.

## Methods

### Design

A retrospective cohort of patients of 60-years-old and over hospitalized for hip fracture during 2002 to 2005 in all public hospitals in 8 Spanish regions. Hospital discharge administrative databases were used to follow up patients from admission to discharge and identify the time to surgical repair and in-hospital mortality.

### Setting

Spain is divided into 17 autonomous regions known as "autonomous communities" with a high degree of self-government, including responsibility for health care. Each Spanish regional government operates an extensive network of hospital and primary healthcare centres that provides free care to about 97% of its respective populations [27]. We use data from 8 autonomous communities (Andalusia, Aragon, Asturias, Basque Country, Valencia Community, Navarre, Galicia and Extremadura) participating in the Spanish Atlas of Medical Practice Variations project [28], a research project emulating the Dartmouth Atlas of Healthcare [29]. Other autonomous communities were not included because their data were not available at the start of the study or had quality problems in some of the study's key variables. The autonomous communities included had around 21.5 million inhabitants in 2005, approximately half of the Spanish population that year.

### Sources of information

The primary source was the so-called "Conjunto Mínimo de Datos Básicos" (Minimum Basic Data Set, MBDS) of hospital discharges produced between 2002 and 2005 in the public hospitals included in the study. The MBDS, a homogeneous register used by all sNHS hospitals, systematically records administrative and clinical data from each hospital discharge with, among other, data about diagnoses and procedures (using the International Classification of Diseases 9th Revision Clinical Modification, ICD9CM), dates of admission, discharge and surgery, and reason for discharge.

### Population

From the 81,740 discharges with a main diagnosis of hip fracture (ICD9CM: 820.xx) produced between January 2002 and December 2005 in the 131 public hospitals in the 8 participating regions, and to ensure a more homogeneous premorbid health status between groups, we excluded 6,889 (8.43%) patients because they were younger than 60-years-old and/or had a diagnosis of multiple or pathological fractures (presumably non-osteoporotic) and/or were elective admissions (suggesting inpatient fractures and that the time from admission to surgery could be not related to the hip surgery) and/or had a length-of-stay longer than 30 days (delays beyond 30 days for non-medical reasons were very unlikely and clinical delays could correspond to very severe patients whose risk differences cannot be adequately adjusted with the available covariates).

**Figure 1:**
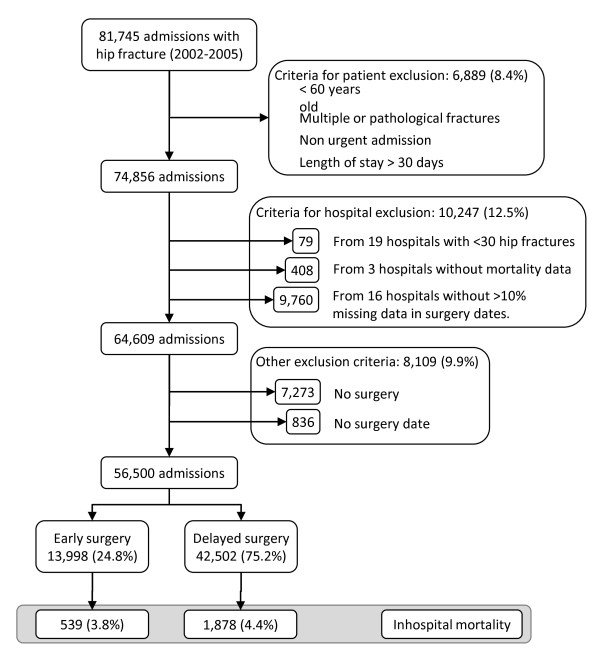
**Study diagram**.

Additionally, we excluded all patients from 19 hospitals (n = 74) with less than 30 hip fracture discharges in the period studied (because we suspect that these hospitals do not have traumatology and orthopaedic wards and cases correspond to patients transferred from other hospitals), 3 hospitals (n = 408 cases) with no deaths registered and 16 hospitals (n = 9760) with more than 10% of discharges without the surgical data recorded (to exclude hospitals with poor quality MBDS registers). From the remaining cases, we excluded patients without the surgical treatment approach (n = 7.273; 11,26%) or without a surgery date (n = 836; 1,02%). The final sample (see Figure 1) included 56,500 patients of 60 years old and over admitted with a hip fracture as a main diagnosis through the emergency department in a public hospital and receiving surgical repair during the episode of care.

### Main outcome measure

In-hospital mortality identified in the discharge criteria codes of the Hospital Discharge MBDS as death or an "in extremis" discharge.

### Main exposure variable

Time to surgery measured as the difference (in days) between the admission date and the surgery date. Because the MBDS does not record the time of admission we were not able to define 24 or 48 hour periods and the exposure variable was grouped as early surgery (repairs that were carried out on the same day or the day following admission) and delayed surgery (repairs that were carried out from the 2nd to the 30th day following admission).

### Other variables and definitions

Variables included in the study that might be associated with the use of early surgery as well as with the probability of death were: age (divided into four categories: 60-69, 70-79, 80-89 and 90-years-old and over), sex, main diagnosis (grouped into three groups: intracapsular fractures (ICD9CM: 820.0x a 820.1x), trochanteric fractures (ICD9CM: 820.2x a 820.3x) and non-otherwise specified fractures (ICD9CM: 820.8 y 820.9), number of secondary diagnoses (grouped into four categories: 0-1, 2-3, 4-6, and > 6), surgical procedure (distinguishing between fixation and arthroplasty), Risk Mortality Index (RMI) modified from the one used by Majumdar et al [15,30] (unfortunately MBDS does not allow differentiation between comorbidities and complications because it does not record the onset of a diagnosis of complications; see Additional File 1 for codes and weights used), Charlson Comorbidity Index calculated following an adaptation of the Charlson Index [31] for administrative databases [32,33]. Our version, as in other Charlson Index adaptations for administrative databases [34,35], distinguishes between codes defining chronic conditions (always accounted for) and acute conditions (those not to be considered in the current admission but in the following admissions; see Additional File 2 for codes and weights used). Finally, the year of discharge (from 2002 to 2005) and whether the admission occurred on weekend or weekdays were also studied.

### Ethics

The databases used in this study were transferred to the researchers without any personal identification. The study, observational and with retrospective non-identifiable data, did not require specific approval by the Ethics and Clinical Trials Committees, or informed consent.

### Analysis

First, we described the characteristics of patients and surgery and a bivariate analysis was carried out (using odds ratios with their respective confidence intervals of 95%, CI95%) to explore the possible associations between these factors and the performance of early-surgery and in-hospital mortality. Second, we used a backward-forward multivariate logistic regression model to analyze the relationship between selected explanatory factors (age, sex, type of fracture, type of surgery, Charlson Comorbidities Index scores, RMI scores and discharge year) and the realization of early surgery. Third, a second backward-forward logistic regression model with mortality as a dependent variable was carried out. We used the same explanatory variables as in the previous model and also our main exposure variable: the timing of surgery. The performance of the models was evaluated using the C-Statistics for discrimination and the Hoshmer-Lemeshow test for calibration.

Additionally, we carried out some complementary analyses: 1) to explore the relationship between the proportion of delayed patients and the in-hospital mortality rate at the hospital level (see Additional File 3), 2) we use multilevel models (a random intercept model adding a random-slope onto early surgery) to explore the impact of the differences between hospitals in the effect of early surgery on mortality (see Additional File 4), and 3) we use an instrumental variable approach (using the day-of-the-week admission as an instrumental variable) to validate the main findings of the study [36] (see Additional File 5).

The analyses were carried out using the STATA^® ^(Statutory, College Station, Texas) statistical package, except for the multi-level analysis that was carried out using R (RDC Team; http://www.R-project.org).

## Results

Two-thirds of the patients were aged 80 and over, with women comprising almost four-fifths of the cases (Table 1). The most common type of fracture was trochanteric (53%), surgical fixation was the most commonly employed repair technique (62%) and 24% of admissions occurred during weekends. The Charlson Comorbidity Index scores were 0 or 1 for 91% of patients and the RMI scores were greater than 6 for nearly 80%. Early surgery was performed on 25% of the patients. Factors significantly associated with higher early surgery in the bivariate analysis include age group (60-69 vs. 70-79 and 80-89), sex (female vs. male), trochanteric fracture (vs. intracapsular fracture), use of fixation techniques (vs. arthroplasty), weekday admission (vs. admission during weekends), lower chronic comorbidity (vs. Charlson index greater than 1), better RMI scores (0 to 6 vs. 7 and over), fewer secondary diagnoses recorded (0-1 vs. 2 and over) and the annual period (frequency of early surgery decreased in 2004 and 2005 vs. 2002).

**Table 1:** Patient characteristics and factors associated with early-surgery.

		All patients		Factors related with early surgery				
		**N**	**%**	**n**^(*)^	**%**	**OR**^(**)^	**95CI OR**	**p**

Age	60-69 years	3,584	6.34	930	25.95	1.00		
	70-79 years	15,840	28.04	3,825	24.15	0.91	0.84-0.99	0.024
	80-89 years	27,309	48.33	6,658	24.38	0.92	0.85-0.99	0.040
	90+ years	9,767	17.29	2,585	26.47	1.03	0.94-1.12	0.547

Sex	Male	12,154	21.52	2,750	22.63	1.00		
	Female	44,328	78.48	11,240	25.36	1.16	1.11-1.22	< 0.001

Fracture	Intracapsular	22,804	40.36	5,400	23.68	1.00		
	Trochanteric	29,999	53.10	7,747	25.82	1.12	1.08-1.17	< 0.001
	NOS	3,697	6.54	851	23.02	0.96	0.89-1.05	0.380

Surgery	Fixation	35,214	62.33	9,394	26.68	1.00		
	Arthroplasty	21,286	37.67	4,604	21.63	0.76	0.73-0.80	< 0.001

Weekend	No	41,759	73.91	10,824	25.92	1.00		
Admission	Yes	14,741	26.09	3,174	21.53	0,78	0,75-0.82	< 0.001

Charlson	0	36,371	64.37	9,644	26.52	1.00		
Index	1	15,222	26.94	3,402	22.35	0.80	0.76-0.83	< 0.001
	2	3,598	6.37	721	20.04	0.69	0.64-0.76	< 0.001
	3	802	1.42	148	18.45	0.63	0.52-0.75	< 0.001
	> 3	507	0.90	83	16.37	0.54	0.43-0.69	< 0.001

Risk	0	1,818	3.22	512	27.98	1.00		
Mortality	4-6	10,367	18.35	2,747	26.33	0.92	0.82-1.03	0.137
Index	7-12	22,237	39.36	5,527	24.70	0.84	0.76-0.94	0.002
	> 12	22,078	39.08	5,212	23.74	0.80	0.72-0.89	< 0.001

Number of	0-1	19,646	34.77	5,783	29.44	1.00		
Secondary	2-3	17,972	31.81	4,629	25.76	0.83	0.80-0.87	< 0.001
Diagnoses	4-6	11,322	20.04	2,364	20.88	0.63	0.60-0.67	< 0.001
	> 6	7,560	13.38	1,222	16.16	0.46	0.43-0.49	< 0.001

Time to	0-1 days	13,998	24.78	13,998	100.00	--	--	--
Surgery	2-5 days	28,069	49.68	--	0.00	--	--	--
	6-14 days	13,303	23.55	--	0.00	--	--	--
	14-30 days	1,130	2.00	--	0.00	--	--	--

Year	2002	13,593	24.06	3,528	25.95	1.00		
	2003	14,403	25.49	3,705	25.72	0.99	0.94-1.04	0.659
	2004	14,233	25.19	3,549	24.94	0.95	0.90-1.00	0.051
	2005	14,271	25.26	3,216	22.54	0.83	0.79-0.88	< 0.001

TOTAL		56,500	100.00	13,998	24.78			

Bivariate analysis.

(*) Number and proportion of patients with early surgery. (**) OR of early surgery regarding the basal value in each stratum. Variables with missing values: sex (n = 18); NOS: Non-Otherwise Specified; OR: odds ratio; 95%CI: 95% confidence interval.

Table 2 shows factors independently associated with early surgery in the multivariate logistic regression analysis. Advanced age (18% more likely to receive early surgery in patients between 80 and 89 or 41% more likely to receive it in patients aged 90 and over), intracapsular fracture (vs. trochanteric) and repair by fixation (vs. arthroplasty) were positively associated with early surgery (23% and 56% more likely, respectively). In turn, the higher the Charlson and RMI scores the lower the probability of receiving early surgery (59% less likely scoring > 3 on the Charlson Index, or 41% less likely ranking over 12 in the RMI score). The last annual period of study (2005) showed a reduction in early surgery, the probability of a patient receiving early surgery being 16% less than in 2002. Nevertheless, the model's discriminative capacity was very low (C-Statistic = 0.56).

**Table 2:** Factors associated with early surgery.

		OR	95%CI OR	p
Age	60-69 years	1.00		
	80-89 years	1.18	1.10; 1.26	< 0.001
	90+ years	1.41	1.25; 1.59	< 0.001

Fracture	Intracapsular	1.00		
	Trochanteric	0.81	0.70; 0.95	0.008

Surgery	Fixation	1.00		
	Arthroplasty	0.64	0.53; 0.77	< 0.001

Charlson	0	1.00		
Index	1	0.82	0.72; 0.93	0.002
	2	0.74	0.61; 0.90	0.002
	3	0.71	0.56; 0.90	0.005
	> 3	0.63	0.50; 0.79	< 0.001

Risk	0	1.00		
Mortality	7-12	0.80	0.74; 0.87	< 0.001
Index	> 12	0.71	0.64; 0.78	< 0.001

Year	2002	1.00		
	2005	0.86	0.76; 0.97	0.016

Multivariate logistic regression analysis.

n = 56482 (18 cases with missing data for the sex variable); r^2 ^= 0.009; p < 0.0001; C-Statistic: 0.565; p(Hoshmer-Lemeshow) = 0.008. Non-significant variables: sex. OR: odds ratio; 95% CI: 95% confidence interval

With regard to in-hospital mortality, 4.3% of patients died (Table 3) during their hospitalization episode, without significant differences between years. In the bivariate analysis, older patients (vs. 60-69 years group), sex (female vs. male), non-specified fractures (vs. intracapsular or trochanteric fractures), higher Charlson Comorbidity index (1 and over vs. 0), higher RMI scores (4 and over vs. 0) and the number of recorded diagnoses (2 and over vs. 0-1) were positively associated with mortality. When it comes to the timing of surgery, interventions performed between 2 and 5 days after admission were not related to higher mortality; however, surgery delayed by more than 5 days was associated with an increasing mortality rate.

**Table 3:** In-hospital mortality related factors.

		All patients	Mortality related factors				
		**n**	n^(*)^	**%**	OR^(**)^	**95%CI OR**	**p**

Age	60-69 years	3,584	37	1.03	1.00		
	70-79 years	15,840	400	2.53	2.48	1.78-3.54	< 0.001
	80-89 years	27,309	1,198	4.39	4.39	3.22-6.00	< 0.001
	90+ years	9,767	782	8.01	8.34	6.02-11.54	< 0.001

Sex	Male	12,154	821	6.75	1.00		
	Female	44,328	1,596	3.60	0.52	0.48-0.56	< 0.001

Fracture	Intracapsular	22,804	898	3.94	1.00		
	Trochanteric	29,999	1,280	4.27	1.09	0.99-1.19	0.070
	NOS	3,697	239	6.46	1.68	1.27-2.23	< 0.001

Surgery	Fixation	35,214	914	4.29	1.00		
	Arthroplasty	21,286	1,503	4.27	0.99	0.92-1.08	0.880

Weekend	No	41,759	1,793	4.29	1.00		
Admission	Yes	14,741	624	4.23	0.98	0.90-1.08	0.755

Charlson	0	36,371	1,144	3.15	1.00		
Index	1	15,222	733	4.82	1.56	1.34-1.80	< 0.001
	2	3,598	324	9.01	3.04	2.59-3.58	< 0.001
	3	802	113	14.09	5.05	3.86-6.61	< 0.001
	> 3	507	103	20.32	7.85	6.07-10.16	< 0.001

Risk	0	1,818	6	0.33	1.00		
Mortality	4-6	10,367	91	0.88	2.39	1.19-6.00	0.017
Index	7-12	22,237	502	2.26	6.97	3.23-15.04	< 0.001
	> 12	22,078	1818	8.23	27.10	12.26-58.42	< 0.001

Number of	0-1	19,646	278	1.42	1.00		
Secondary	2-3	17,972	486	2.70	1.94	1.51-2.48	< 0.001
Diagnoses	4-6	11,322	614	5.42	3.99	3.00-5.31	< 0.001
	> 6	7,560	1,039	13.74	11.10	8.15-15.13	< 0.001

Time to	0-1 days	13,998	539	3.85	1.00		
Surgery	2-5 days	28,069	1,070	3.81	0.98	0.88-1.11	0.860
	6-14 days	13,303	696	5.23	1.38	1.17-1.61	< 0.001
	14-30 days	1,130	112	9.91	2.75	2.01-3.75	< 0.001

Year	2002	13,593	606	4.46	1.00		
	2003	14,403	641	4.45	0.99	0.87-1.14	0.980
	2004	14,233	594	4.17	0.93	0.81-1.06	0.298
	2005	14,271	576	4.04	0.90	0.79-1.03	0.135

TOTAL		56,500	2,417	4.28			

(*) Number and proportion of dead patients. (**) OR of death regarding the basal value in each stratum. Variables with missing values: sex (n = 18); NOS: Non-Otherwise Specified; OR: odds ratio; 95%CI: 95% confidence interval.

Table 4 shows absolute differences in mortality between early and delayed surgery stratified by the above-mentioned variables. In this non-adjusted analysis, early surgery (as opposed to delayed surgery) had a statistically significant absolute reduction of 0.57% (from 4.42% to 3.85%) in in-hospital mortality. This protective association was also found in some of the stratification groups (some age groups, women, intracapsular fractures, arthroplasty repair, weekday admission, and the year 2000) but not noticeably in others. Thus, no differences in mortality between early and delayed surgery were found in the same stratum of the Charlson Index, RMI or number of secondary diagnoses (except in Charlson scores > 6, but favouring delayed surgery).

**Table 4:** Mortality differences between early and delayed surgery.

		Mortality in early surgery		Mortality in delayed surgery		Mortality differences between early and delayed surgery		
		**N**	**%**	**n**	**%**	**%**	**95CI**	**p**

Age	60-69 years	930	0.86	2,654	1.09	-0.23	-0.95; 0.48	0.546
	70-79 years	3,825	2.09	12,015	2.66	-0.57	-1.10; -0.03	0.050
	80-89 years	6,658	3.97	20,651	4.52	-0.56	-1.10; -0.01	0.053
	90+ years	2,585	7.23	7,182	8.28	-1.05	-2.24; 0.13	0.091

Sex	Male	2,750	6.58	9,404	6.81	-0.22	-1.28; 0.83	0.681
	Female	11,240	3.19	33,088	3.84	-0.56	-0.94; -0.17	0.006

Fracture	Intracapsular	5,400	3.31	17,404	4.13	-0.82	-1.38; -0.25	0.007
	Trochanteric	7,747	3.95	22,252	4.38	-0.43	-0.94; 0.08	0.109
	NOS	851	6.35	2,846	6.50	-0.15	-2.02; 1.72	0.872

Surgery	Fixation	9,394	3.94	25,820	4.47	-0.45	-0.92; -0.02	0.065
	Arthroplasty	4,604	3.67	16,682	4.39	-0.79	-1.42; -0,17	0.018

Weekend	No	10,824	3.73	30,935	4.49	-0.76	-1.18; -0.33	< 0.001
Admission	Yes	3,174	4.25	11,567	4.23	0.02	-0,77; 0.82;	0.949

Charlson	0	9,644	2.98	26,727	3.21	-0.23	-0.63; 0.17	0.266
Index	1	3,402	4.32	11,820	4.96	-0.64	-1.42; 0.15	0.126
	2	721	8.88	2,877	9.04	-0.16	-2.49; 2.16	0.893
	3	148	18.92	654	13.00	5.92	-0.89; 12.73	0.061
	> 3	83	15.66	424	21.23	-5.56	-14.30; 3.17	0.249

Risk	0	512	0.00	1,306	0.46	-0.46	-0.83; -0.09	0.124
Mortality	4-6	2,747	0.66	7,620	0.96	-0.30	-0.67; 0.06	0.145
Index	7-12	5,527	2.12	16,710	2.30	-0.19	-0.63; 0.02	0.417
	> 12	5,212	7.75	16,866	8.38	-0.63	-1.47; 0.02	0.147

Number of	0-1	5,783	1,25	13,863	1.49	-0.24	-0.59; 0.10	0.193
Secondary	2-3	4,629	3.05	13,343	2.59	-0.49	-0.10; 1.02	0.096
Diagnoses	4-6	2,364	5.58	8,958	5.38	0.20	-0.83; 1.23	0.698
	> 6	1,222	15.88	6,338	13.33	2.54	0.33; 4.75	0.018

Time to	0-1 days	13,998	3.85	--	--	--	--	--
Surgery	2-5 days	--	--	28,069	3.81	--	--	--
	6-14 days	--	--	13,303	5.93	--	--	--
	14-30 days	--	--	1,130	9.91	--	--	--

Year	2002	3,528	3.29	10,065	4.87	-1.58	-2.30; -0.86	< 0.001
	2003	3,705	3.99	10,698	4.61	-0.61	-1.36; 0.13	0.118
	2004	3,549	4.25	10,684	4.15	0.10	-0.66; 0.87	0.780
	2005	3,216	3.86	11,055	4.09	-0.23	-0.99; 0.53	0.555

TOTAL		13,998	3.85	42,502	4.42	-0.57	-0.94; -0.19	0.004

Stratified analysis.

Variables with missing values: sex (n = 18); NOS: Non-Otherwise Specified; 95%CI: 95% confidence interval.

Finally, Table 5 shows factors independently associated with in-hospital mortality in the multivariate logistic regression analysis. Age (the older the more likely), men (being male increases the probability of death by 16%), suffering a "non-specified" fracture (67% more likely to die), higher scores in the Charlson Index (up to 5.4 times more likely to die if the Charlson Index ranking is 3 or more) and RMI score (up to 6.4 times more likely to die if RMI scores are 12 or more) were independent factors associated with higher mortality. The type of surgery and the timing of surgery were not associated with in-hospital mortality after adjusting for the other variables in the model. Adjusted mortality was seen to decrease over the study period. The model showed a moderate discriminative power (C-Statistic: 0.74), whereas calibration was weak (p Hoshmer-Lemeshow test = 0.006).

**Table 5:** Factors associated with mortality.

		OR	95CI OR	p
Age	60-69 years	1.00		
	70-79 years	1.61	1.12; 2.30	0.009
	80-89 years	2.11	1.47; 3.03	< 0.001
	90+ years	2.40	1.63; 3.54	< 0.001

Sex	Man	1.00		
	Woman	0.89	0.80; 0.99	0.032

Fracture	Intracapsular	1.00		
	NOS	1.67	1.27; 2.19	< 0.001

Charlson	0	1.00		
index	1	1.49	1.29; 1.72	< 0.001
	2	2.49	2.13; 2.91	< 0.001
	3	3.36	2.55; 4.43	< 0.001
	> 3	5.37	4.09; 7.04	< 0.001

Risk	0	1.00		
mortality	7-12	2.02	1.59; 2.59	< 0.001
index	> 12	6.38	4.85; 8.38	< 0.001

Year	2002	1.00		
	2004	0.90	0.81; 1.00	0.052
	2005	0.84	0.74; 0.95	0.005

Multivariate logistic regression analysis.

n = 56482 (18 cases with missing data for the sex variable); r^2 ^= 0.095; p < 0.0001; C-Statistic: 0.745; p(Hosmer-Lemeshow) = 0.006. Non-significant variables: Type of surgery (arthroplasty vs. internal fixation), time to surgery (early vs. delayed). OR: odds ratio; 95CI: 95% confidence interval.

In the complementary analysis, 1) the hospital-aggregated proportion of late surgery was not linearly related with the hospital-aggregated mortality in patients operated on for hip fracture (Additional File 3); 2) in the multi-level analysis, around 2% of the variance in risk mortality would be attributable to the hospital level (with 13 hospitals significantly over or under the expected mortality) but we did not find evidence that the effect of delayed surgery on mortality was different across hospitals (Additional File 4) the instrumental variable approach using the admission day-of-the-week as an instrumental variable did not affect the (lack of) relationship between early surgery and in-patient mortality in the adjusted analysis (Additional File 5).

## Discussion

This cohort study on the relationship between early repair of hip fracture and in-hospital mortality draws out an (unadjusted) absolute difference in the risk of mortality of 0.57 (95%CI: 0.19 to 0.94) favouring early surgery over delayed. However, patients undergoing delayed surgery were older and had higher severity as measured by the Charlson and the RMI indexes and, therefore, a higher mortality risk. Timeliness on surgery was not found to be related to mortality once confounding factors such as age, sex, chronic comorbidities as well as severity of illness were controlled for in the multivariate analysis. Complementary analysis at hospital aggregated level, multi-level analysis and the instrumental variable approach do not alter these results, which are consistent with most of the studies that have used risk adjustment measures to determine whether patient condition, as opposed to the organization of care, explained the variation in death.

One interesting observation that underpins the idea that other factors, rather than timeliness, are involved in achieving good outcomes after hip fracture surgery is the noticeable decrease in the rate of early surgery over time (a 16% relative reduction in the study timeframe, with an increase of 1 day in the pre-operative length-of-stay), together with a 19% relative decrease in the in-hospital mortality rate. In the same way, a recent study compared the management of hip fracture in Japanese and USA hospitals showing that the length of preoperative stay was very different (1 day in the USA vs. 5 days in Japan) but one-year mortality was similar in both countries [26]. The hypothesis that avoidable mortality in hip fracture is determined by a combination of factors would be consistent with the findings of a recent nationwide cohort study aimed at describing the effect of the quality of care on 30 day mortality after hip-fracture. The study draws out a dose-response effect of being 5.6 times less likely to die if all the 5 agreed quality criteria (as opposed to none) were properly accomplished [37].

The simultaneous decreasing trends in the practice of early surgery and mortality rates together with the poor discrimination of the regression model on early vs. delayed surgery (Table 3) suggest that unobserved factors related to the care itself are responsible for better results. In fact, one of the better practices throughout Spain in recent years has been the use of tromboembolic prophylaxis, as well as the administration of anti-coagulant drugs or early mobilization. Indirect evidence from a nationwide study in Spain showed a 28% relative reduction in pulmonary tromboembolism and deep venous thrombosis events in hip fracture patients between 2002 and 2005 [38], underpinning the idea of other interventions rather than the decision on early surgery being behind better results. Organizational changes in the period such as the introduction of integrated clinical pathways [39,40], various forms of geriatric or medical co-management [41,42] and a better knowledge of hip fracture syndrome may also have contributed to the decreasing mortality trend.

On the other hand, the surgical alternatives (fixation vs. arthroplasty) which remain in the model as an independent factor in the decision to perform early surgery, together with the already mentioned limited capacity of the model to explain that decision, suggests that the surgical decision process is based on more subtle variables than those used to build the regression model. The impact of this lack of information appears not to affect our results because intervention with one or the other surgical alternative did not affect the risk of death in our sample, but the topic is relevant because different criteria for selecting patients for early or delayed surgery, including surgery modality, could change the relationship between the timing of surgery and mortality. Orthopaedic surgeons and anaesthesiologists use certain, though not always well-defined criteria, to select patients for early or delayed surgery, and it is possible that different subgroups of patients could benefit from one or other surgical strategy.

### Limitations

First of all, our study was observational, retrospective and used administrative databases as the source of information. While criteria for inclusion tried to homogenize the sample and minimize data quality problems, the shortcuts and pitfalls of this data for risk-adjustment are well known [43]. Regarding the main diagnosis, a recent study in the Netherlands has shown that hip fracture may be miscoded by up to the 3% in administrative databases [44]. The paucity of secondary diagnoses recorded (two-third of patients with 3 or less) and the high volume of patients with a Charlson score equal to 0 (our sample only included elderly people, more than 50% over 80 years-old) warns about the possibility of information biases that might affect risk-adjustment. Although Spanish regional departments of health have carried out some internal audits on the Minimum Basic Data Sets in their respective regions, there are very few published studies on data quality in Spain (to our knowledge none of them auditing the hip fracture coding) and its results show problems of accuracy, completeness and information biases [45-47] which are expected to have affected the risk adjustment analysis in some way. Nonetheless, and for the main objective of our study, these problems would suppose an important bias only if there was differential between early or delayed surgery groups and/or with the main study endpoint. As observed in the stratified analysis (Table 4), differences in mortality according to coding strata were not significant, except in the case of patients with more than 6 secondary diagnoses where the death rate was higher in early surgery (a 2.54% absolute difference).

Also related to information biases is the fact that the MBDS do not provide chronological information about secondary diagnoses. While diagnoses for chronic conditions can be confidently treated as comorbidities, acute conditions could be comorbidities or complications. The inability to distinguish among them, together with the possibility of a selective recording of a severe diagnosis depending on the evolution of the patient, reduces the capacity to properly adjust for individual risk. The effect on the estimated differences would be expected to be small, since no differences in mortality were found between any one of the strata, either in the Charlson or RMI Indexes (Table 4). However, the fact that some recorded complications could be either the cause or consequence of delayed surgery does not allow the ruling-out of biased estimates on mortality in the case of this surgical approach. These information biases could tend to overadjust cases with poor outcomes and to attenuate (if they really exist) mortality differences between early and delayed surgery.

The use of inpatient death as the main endpoint (vs. mortality within a fixed time, such as 30 days or one-year from admission, data not available in our study) involves several inconveniences [48]. More aggressive discharge policies can reduce the "mortality" rates if patients are discharged "alive" to die at home. On the contrary, a longer length of stay (LOS) increases the probability of identifying some outcomes such as patient mortality. Because delayed surgery is associated with longer LOS the mortality in this group is probably overemphasized. If poor patient condition was associated with the decision to delay the intervention then these problems could also contribute to overstating mortality in this group. Additionally, the recording of a secondary diagnosis could be associated with LOS and poor outcomes, also affecting the covariates adjustment. The available endpoint does not permit the overcoming of these problems that could bias our results if different discharge policies are used in patients with poor outcomes (associated with one or another of the surgical alternatives evaluated).

One limitation that could potentially affect the estimation of non-differences between early and delayed surgery is the lack of information on the probability of a patient dying in the days immediately following admission. Moreover, the observed cutback in the total length of stay (one day, in spite of the increase in pre-operative stays) over the follow-up period may increase the risk of bias. If we could assume that mortality after discharge followed a random distribution, the estimates would not be affected. We have favoured this possibility by adding "in extremis" patients (i.e. patients who are discharged to die at home) to the case definition, but we are not able to rule out that the hospitals, in our sample, discharge patients depending on the risk of short-term death, thus affecting the non-difference on the timing of surgery.

Finally, the inclusion of only 8 autonomous communities could also be a source of bias if the non-included communities showed different behaviour. Nevertheless, the participating autonomous communities are quite diverse and include practically half of the Spanish population.

### Implications

Late hip fracture surgery could be due to different reasons. Unavoidable delays in more severe patients unfit for surgery, better control of hemorrhagic risks in patients taking oral anticoagulants or antiplatelet agents, and organizational problems are the most important, being problems caused by the shortage of orthopaedic surgeons or anaesthesiologists very unusual in Spain. With some uncertainty derived from the limitations stated, our results do not show any independent association between early surgery and reduced mortality and does not support the use of early surgery as a quality indicator related with poor outcomes in the Spanish NHS setting. Anyway, and independently of its impact on mortality, while early surgery could shorten the length of stay and its more comfortable for patients, we found a huge variation in the proportion of "early surgery" between hospitals and a surprisingly low average of "early surgery" compared to international standards. These data suggest the need for quality improvement in Spain in terms of good practices in identifying patients that can (or cannot) be operated early. On the other hand, and beyond the delays for organizational reasons, orthopaedic surgeons and/or anaesthesiologists seem to use specific criteria for delaying certain patients. Probably, before developing new studies that analyse the effectiveness of early vs. delayed surgery in all hip fracture patients regardless their clinical status, new research projects should be directed to identify patients with more probability of benefitting from the timing of each surgical approach in the actual context of care for hip fracture patients.

## Conclusions

In-hospital mortality in older patients with hip fractures is about 4.3% in the Spanish National Health System. Older age, male, higher chronic comorbidity and higher severity measured by the RMI were associated with higher mortality, but the timing of surgery was not. These results are observational in nature and therefore applicable to the actual criteria employed in our setting to select patients for early or delayed surgery.

## Competing interests

The authors declare that they have no competing interests.

## Authors' contributions

JL, SP and EBD are guarantors of the study, had full access to all the data, and take responsibility for the integrity and the accuracy of the analysis and results. All authors contributed to the conception and the design of the article. NML acted as data-manager for the study. JL, SP, EL and GSG contributed to the study analysis. JL, SP, EBD and JM interpreted the results, and SP, GSG and EBD drafted the article. All the authors read and approved the final manuscript.

## Pre-publication history

The pre-publication history for this paper can be accessed here:

http://www.biomedcentral.com/1472-6963/12/15/prepub

## Supplementary Material

Additional file 1 **Charlson Index**. adaptation of the Charlson Comorbidity index for administrative databases. Click here for file

Additional file 2 **Risk Mortality Index**. adaptation of the Majumdar et al. Risk Mortality Index for hip fracture. Click here for file

Additional file 3 **Hospital level approach**. Scatter plot between the proportion of delayed patients by hospital and the inhospital mortality rate. Click here for file

Additional file 4 **Multilevel approach**. Variations in in-hospital mortality between hospitals. A multi-level approach. Click here for file

Additional file 5 **Instrumental variable approach**. Instrumental variable analysis. Click here for file
