# Supplementary material for: Timing of surgery for hip fracture and in-hospital mortality: a retrospective population-based cohort study in the Spanish National Health System
Source: BMC Health Serv Res. 2012 Jan 18;12:15. doi: 10.1186/1472-6963-12-15 (PMC3292938; doi:10.1186/1472-6963-12-15)
Supplement: Additional file 1 — Charlson Index. adaptation of the Charlson Comorbidity index for administrative databases. [file 1472-6963-12-15-S1.DOC]

**Timing of surgery for hip fracture and in-hospital mortality. A retrospective population-based cohort study in the Spanish National Health System.**

**Additional File 1.**

Adaptation of the Charlson Comorbidity Index for administrative databases

| **DIAGNOSIS CATEGORY** | **ICD9CM CODES** | **WEIGHT** |
| --- | --- | --- |
| Myocardial infarction | 410.xx, 412^*^ | 1 |
| Congestive heart failure | 402.01, 402.11, 402.91, 404.01, 404.03, 04.11,404.93 425.x, 428.x, 429.3 | 1 |
| Periferical vascular disease | 440.x^*^, 441.x^*^, 442.x^*^, 443.1-443.9^*^, 447.1^*^, 785.4^*^, V43.4^*^, 38.14^P*^, 38.16^P*^, 38.18^P*^, 38.33-38.34^P*^, 38.36^P*^, 38.38^P*^, 38.43-38.44^P*^, 38.46^P*^, 38.48^P*^, 39.22-39.26^P*^, 39.29^P*^ | 1 |
| Cerebrovascular disease | 362.34, 430-436, 437-437.1, 437.9, 438^*^, 781.4, 784.3, 997.0, 38.12^P^, 38.42^P^ | 1 |
| Dementia | 290.x^*^, 331-331.2^*^ | 1 |
| Chronic lung disease | 415.0^*^, 416.8-416.9^*^, 490-496^*^, 500-505^*^, 506.4^*^ | 1 |
| Rheumatological diseases | 710.0-710.1^*^, 710.4^*^, 714.0-714.2^*^, 714.81^*^, 725^*^ | 1 |
| Peptic ulcer disease | 531.0-531.3, 531.4x-531.7x^*^, 531.9, 532.0x-532.3x, 532.4x-532.7x^*^, 532.9, 533.0x-533.3x, 533.4x-533.7x^*^, 533.9, 534.0x-534.3x, 534.4x-534.7x^*^, 534.9 | 1 |
| Slight hepatic diseases | 571.2^*^, 571.4^*^, 571.5-571.6x^*^, 571.8-571.9^*^ | 1 |
| Slight or moderate diabetes | 250.0x-250.3x^*^ | 1 |
| Diabetes with chronic complications | 250.4x-250.9^*^ | 1 |
| Hemiplegia o paraplegia | 342.x^*^, 344.0, 344.1^*^, 344.2-344.9 | 2 |
| Kidney disease | 582.x^*^, 583.0-583.7^*^, 585-586^*^, 588.x^*^, V42.0^*^, V45.1^*^, V56.x^*^, 39.27^P*^, 39.42^P*^, 39.93-39.95^P*^, 54.98^P*^ | 2 |
| Malignant neoplasias, including lymphomas and leukaemias | 200.xx-208.xx^*^, 273.0^*^, 273.3^*^, V10.46^*^, 60.5^P*^, 62.4-62.41^P*^ | 2 |
| Moderate or severe liver disease | 572.2-572.8^*^, 456.0-456.2x^*^, 39.1^P*^, 42.91^P*^ | 3 |
| Solid tumour metastasis | 196.x-199.x^*^ | 6 |
| Acquired immunodeficiency syndrome | 042.x-044.x | 6 |
| *Codes not included if they appear in the index episode as the main diagnosis, but are included if they were noted as secondary. The rest of the codes, and also the former one, are included when they existed in prior episodes, and were diagnosed as main or secondary. The asterisk is applied to all codes within each range. ^P^ICD9CM procedure codes. ICD9CM: International Classification of Diseases 9^th^ revision Clinical Modification. | | |
