# Supplementary material for: Timing of surgery for hip fracture and in-hospital mortality: a retrospective population-based cohort study in the Spanish National Health System
Source: BMC Health Serv Res. 2012 Jan 18;12:15. doi: 10.1186/1472-6963-12-15 (PMC3292938; doi:10.1186/1472-6963-12-15)
Supplement: Additional file 2 — Risk Mortality Index. adaptation of the Majumdar et al. Risk Mortality Index for hip fracture. [file 1472-6963-12-15-S2.DOC]

**Timing of surgery for hip fracture and in-hospital mortality. A retrospective population-based cohort study in the Spanish National Health System.**

**Additional File 2.**

Adaptation of the Majumdar et al., Risk Mortality Index

| **VARIABLES** |  | **ICD9CM CODES** | **WEIGHT** |
| --- | --- | --- | --- |
| Diagnoses | Myocardial infarction | 410.x, 411.x | 13 |
|  | Heart Failure | 428.x | 7 |
|  | Cardiac Arrhytmias | 426.x^*^, 427.x | 5 |
|  | Ischemic heart disease | 412.x-414.x | 14 |
|  | Chronic Obstructive Pulmonary Disease | 490.x-496.x | 4 |
|  | Pneumonia | 480.x-486.x | 14 |
|  | Renal failure | 584.x-586.x | 19 |
|  | Electrolytes abnormal | 276.x | 5 |
|  | Malnutrition | 261.x-263.x | 20 |
|  | Malignancy | 140.x-208.x | 13 |
| Other | Age | 60-69 | 0 |
|  |  | 70-79 | 6 |
|  |  | 80-89 | 7 |
|  |  | >89 | 13 |
|  | Male gender |  | 6 |
| The original index includes the variable “Admitted from long-term care”, which is not available in Spanish hospital administrative databases. Diagnoses selected using the corresponding ICD9CM codes. ICD9CM: International Classification of Diseases 9^th^ revision Clinical Modification codes. | | | |
