# Supplementary material for: Timing of surgery for hip fracture and in-hospital mortality: a retrospective population-based cohort study in the Spanish National Health System
Source: BMC Health Serv Res. 2012 Jan 18;12:15. doi: 10.1186/1472-6963-12-15 (PMC3292938; doi:10.1186/1472-6963-12-15)
Supplement: Additional file 3 — Hospital level approach. Scatter plot between the proportion of delayed patients by hospital and the inhospital mortality rate. [file 1472-6963-12-15-S3.DOC]

**Timing of surgery for hip fracture and in-hospital mortality. A retrospective population-based cohort study in the Spanish National Health System.**

**Additional File 3.**

Scatter plot between the proportion of delayed patients by hospital and the inhospital mortality rate.

Sund et al [1,2] proposed the simultaneous examination of the provider level proportions of late surgery patients and mortality to estimate the association between a larger share of delayed surgery and mortality at hospital level.

Because there is a huge variation between hospitals in the proportion of patients operated on with delay (see Figure 1) we can expect that the percentage of late surgery patients could be reduced to a potentially achievable level (the percentage in hospitals with a lower proportion of delayed surgery, lower than 10% in some hospitals in our study). This level, following Sund’s interpretation [2], can be assumed as the upper limit for the proportion of acceptably delayed patients and, correspondingly, the expected proportion of unacceptably delayed patients is the proportion of late surgery patients exceeding this upper limit.

Under these assumptions, we can expect that the overall hospital mortality for patients operated on for hip fracture will increase with an increase in the proportion of delayed patients if longer operative delays are associated with higher mortality. Therefore, the association between delayed surgery and mortality could be analyzed by estimating trends between the proportions of delayed patients and mortality by hospital.

Figure 1 shows a scatter plot between proportions of delayed patients and mortality. Trends were estimated using a linear OLS regression (green line), and a smoother estimation by means of a polynomial regression (red line). As expected, in the linear OLS regression, the overall non-adjusted hospital level mortality of hip fracture patients increases (discreetly) with an increasing proportion of delayed surgery patients (r^2^ = 0,0102), but the trend was not significant (p=0.3357). In the smoother polynomial regression, mortality was non-linearly associated with the proportion of delayed surgery patients, with a sharp decline in mortality after exceeding percentages around 80-85% of patients with delayed surgery.

Because hospitals when (nearly) all patients who are delayed have mortality rates which are very similar to hospitals in the lower level of surgery delays, results suggest that the effect on mortality of the delay in operating is mainly due to unavoidable delays with more severe patients who are unfit for surgery (and not to the delay itself).

1. Sund R, Liski A: Quality effects of operative delay on mortality in hip fracture treatment. Qual Saf Health Care 2005, 14(5):371-7.

2. Sund R. Further evidence from routine data by using the hospital-level hypotheses. BMJ rapid responses. 2006; http://www.bmj.com/content/332/7547/947/reply

| **Additional file 3 - Figure 1. Scatter plot between proportions of delayed patients and mortality by hospital.** |
| --- |
| **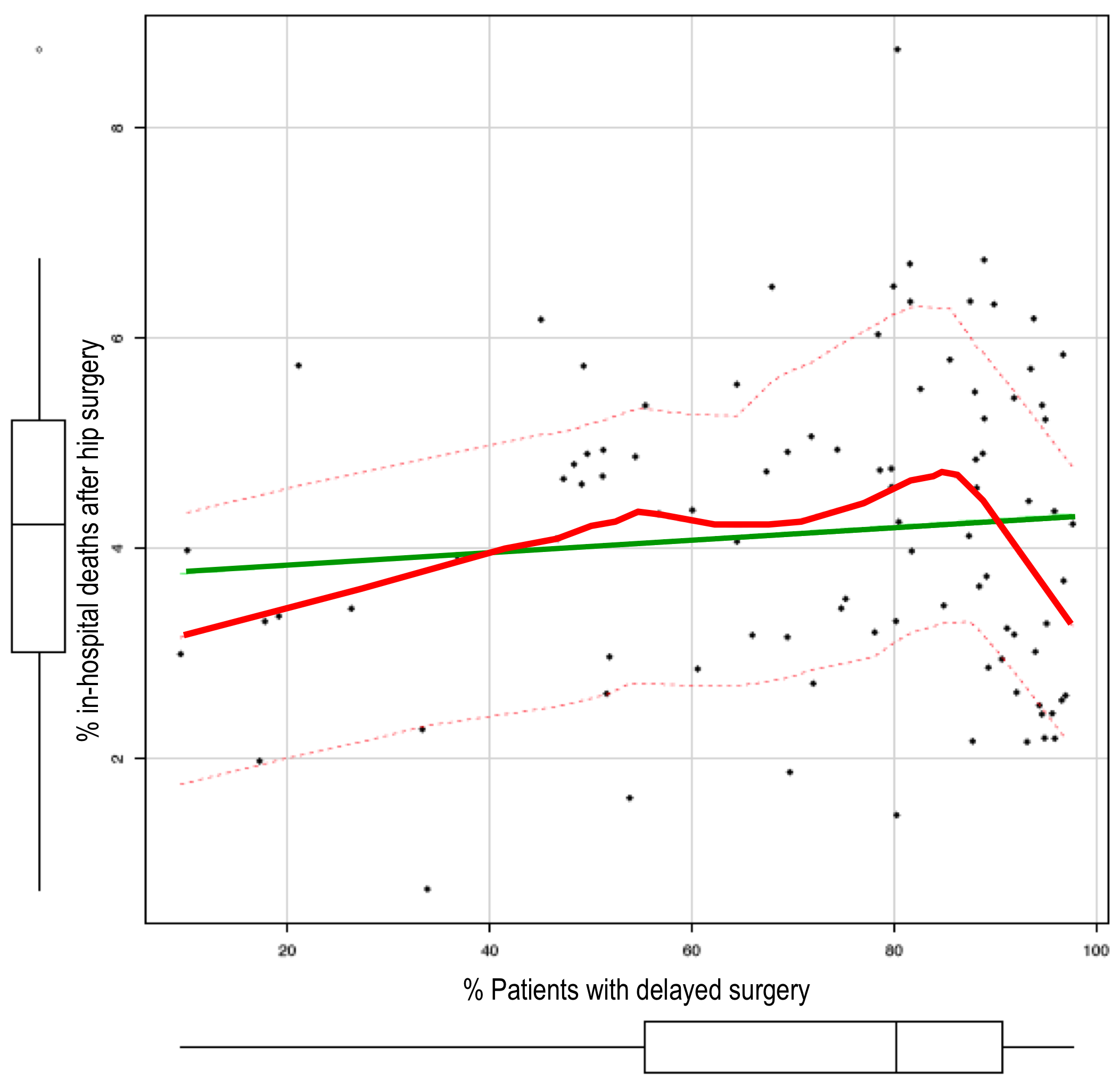** |
| Green line: linear regression OLS estimated; Red Line: Smoother polynomial regression. |
