# Supplementary material for: Timing of surgery for hip fracture and in-hospital mortality: a retrospective population-based cohort study in the Spanish National Health System
Source: BMC Health Serv Res. 2012 Jan 18;12:15. doi: 10.1186/1472-6963-12-15 (PMC3292938; doi:10.1186/1472-6963-12-15)
Supplement: Additional file 4 — Multilevel approach. Variations in in-hospital mortality between hospitals. A multi-level approach. [file 1472-6963-12-15-S4.DOC]

**Timing of surgery for hip fracture and in-hospital mortality. A retrospective population-based cohort study in the Spanish National Health System.**

**Additional File 4.**

Variations in in-hospital mortality between hospitals. A multi-level approach.

The association between the proportion of early surgery and in-hospital mortality could be different between hospitals. We use a multi-level approach: 1) to determine the variation between hospitals in the risk of mortality after hip fracture surgery using a multilevel logistic random intercept model; and 2) to explore the effect (constant versus heterogeneous) of early surgery on mortality across hospitals using a multilevel logistic random coefficient model.

We use multilevel models with the set of covariates reported as significant in Table 5 from the article (age, sex, fracture, Charlson index, risk mortality index and year). First, we analyze the variation in the mortality risk between hospitals contrasting the hypothesis of no differences and otherwise quantifying its importance through the variance partition coefficient obtained by the latent method. It is assumed that the effect of risk factors is constant (the same gradient) across hospitals. Second, we contrast the hypothesis that the (lack of) effect of delayed surgery over mortality is constant across hospitals, using a model with a random coefficient for this implemented variable. To carry out these analyses, a series of models and contrasts between them were estimated:

In the first model (M1), we replicate the multivariate logistic regression shown in Table 5 in the article, but using the enter method (instead of the backward-forward method) to retain all variables, including non-significant variables. **Table 1** and **Table 2** of this additional file show the coefficients of the M1 regression in log odds and odds-ratio scales.

| **Additional file 4 - Table 1. Factors associated with mortality. Multivariate logistic regression analysis (enter method) in log odds scale (M1 model).** |
| --- |
| **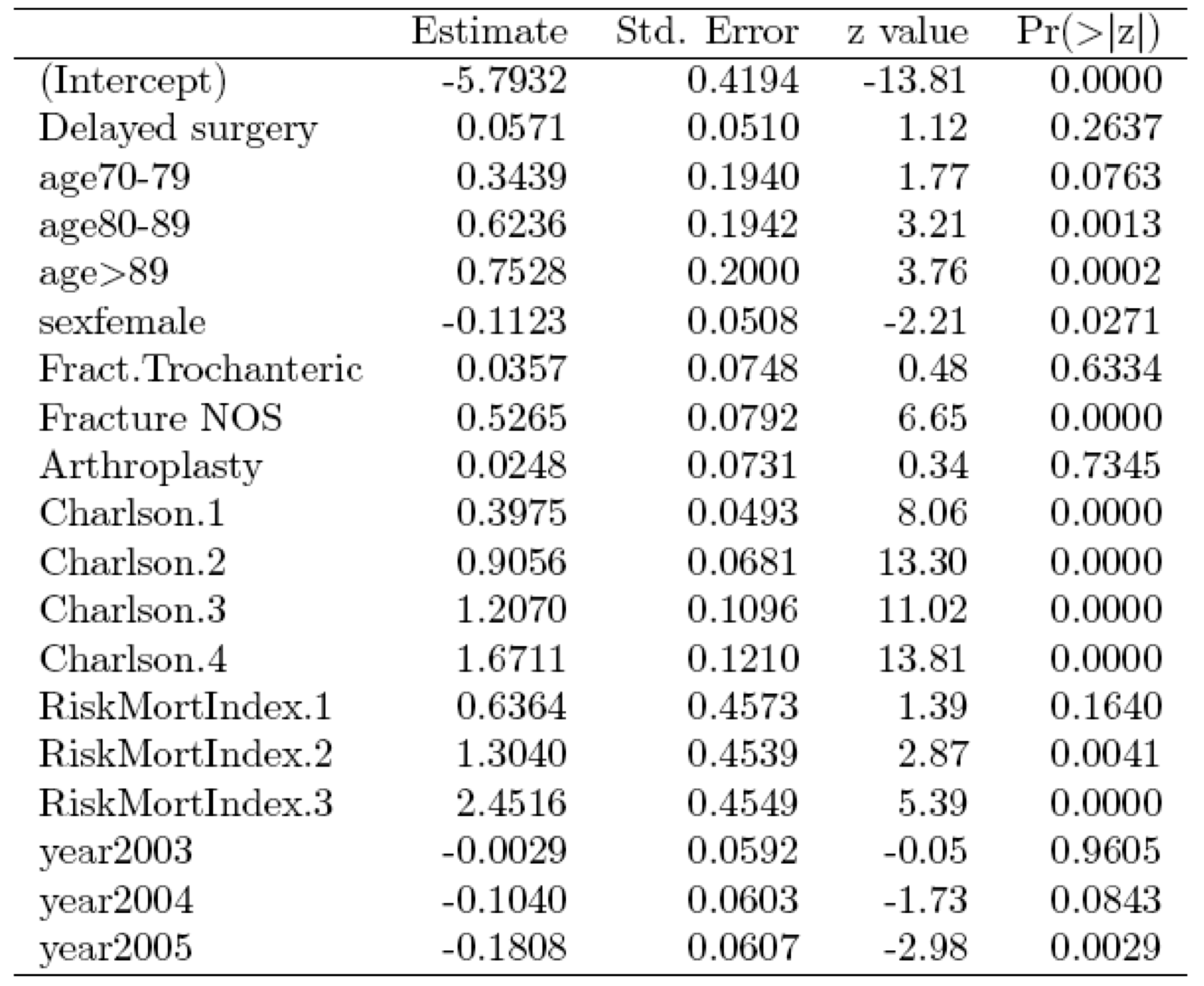** |

| **Additional file 4 - Table 2. Factors associated with mortality. Multivariate logistic regression analysis (enter method) in odds ratio scale (M1 Model).** |
| --- |
| **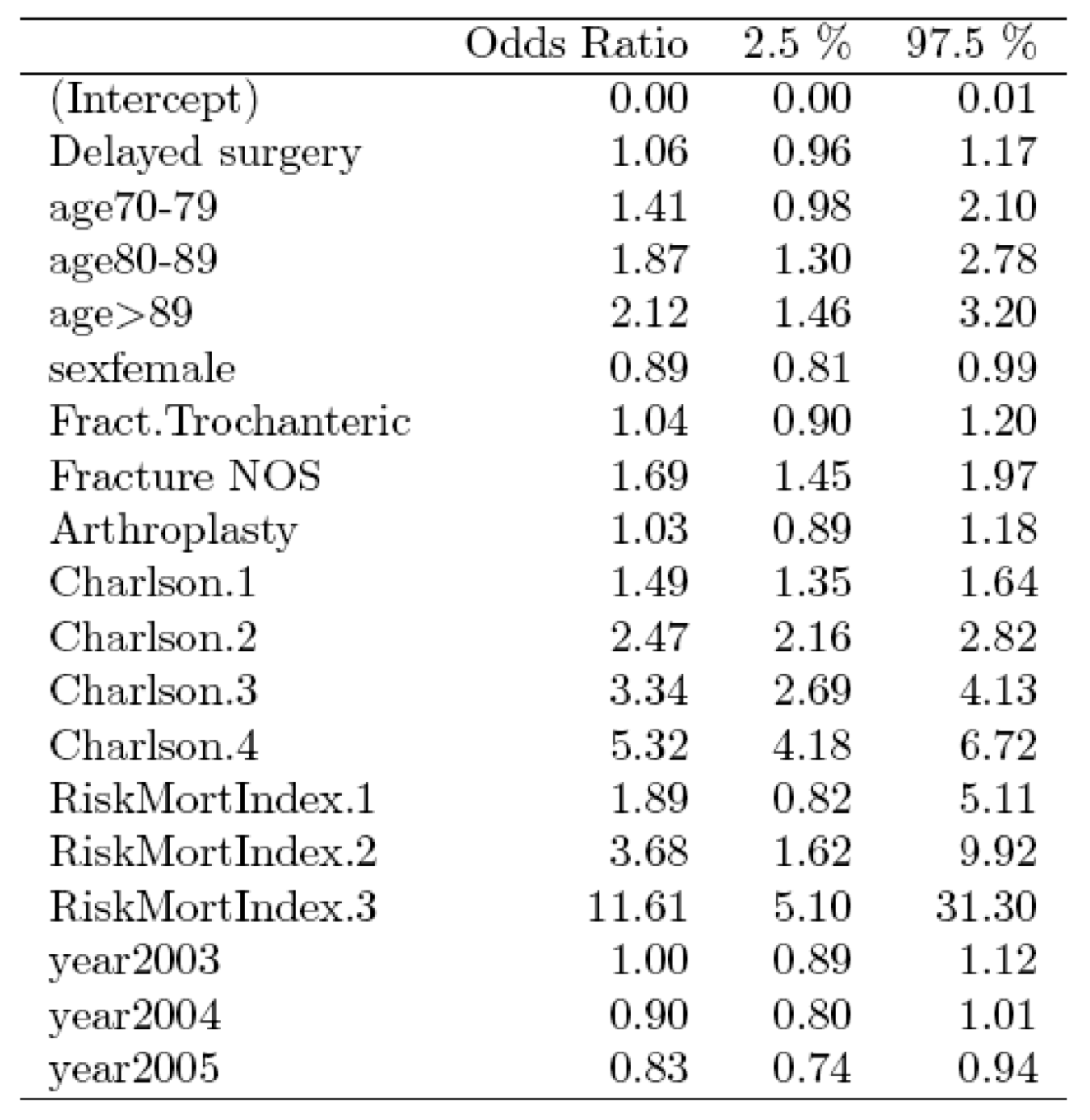** |

Second, we estimate a multilevel logistic random intercept model in order to measure the variability between hospitals and the effect attributable to this level of aggregation, using the Laplace (M2L) and the Markov Chain Monte Carlo (M2M) approaches. **Table 3** shows the fixed-effect part of the M2L model and **Figure 1** the estimated residuals (M2M) for all 93 hospitals in the sample using this random-intercept model. In 13 hospitals the 95% credible interval does not overlap the horizontal line at zero (variance partition coefficient CI 1.41; 3.74%) with a DIC value of 17933.12. Therefore, around 2% of the risk mortality variability would be attributable to hospital level and the mortality level in 13 hospitals is over or under the expected one. The hospital effect was tested contrasting the M1 and M2L models with the log likelihood ratio (LR) test. The LR test value was 95.29 (p<0.0001) rejecting the hypothesis of no hospital effect.

| **Additional file 4 - Table 3. Multilevel logistic random intercept model (M2L Model).** |
| --- |
| **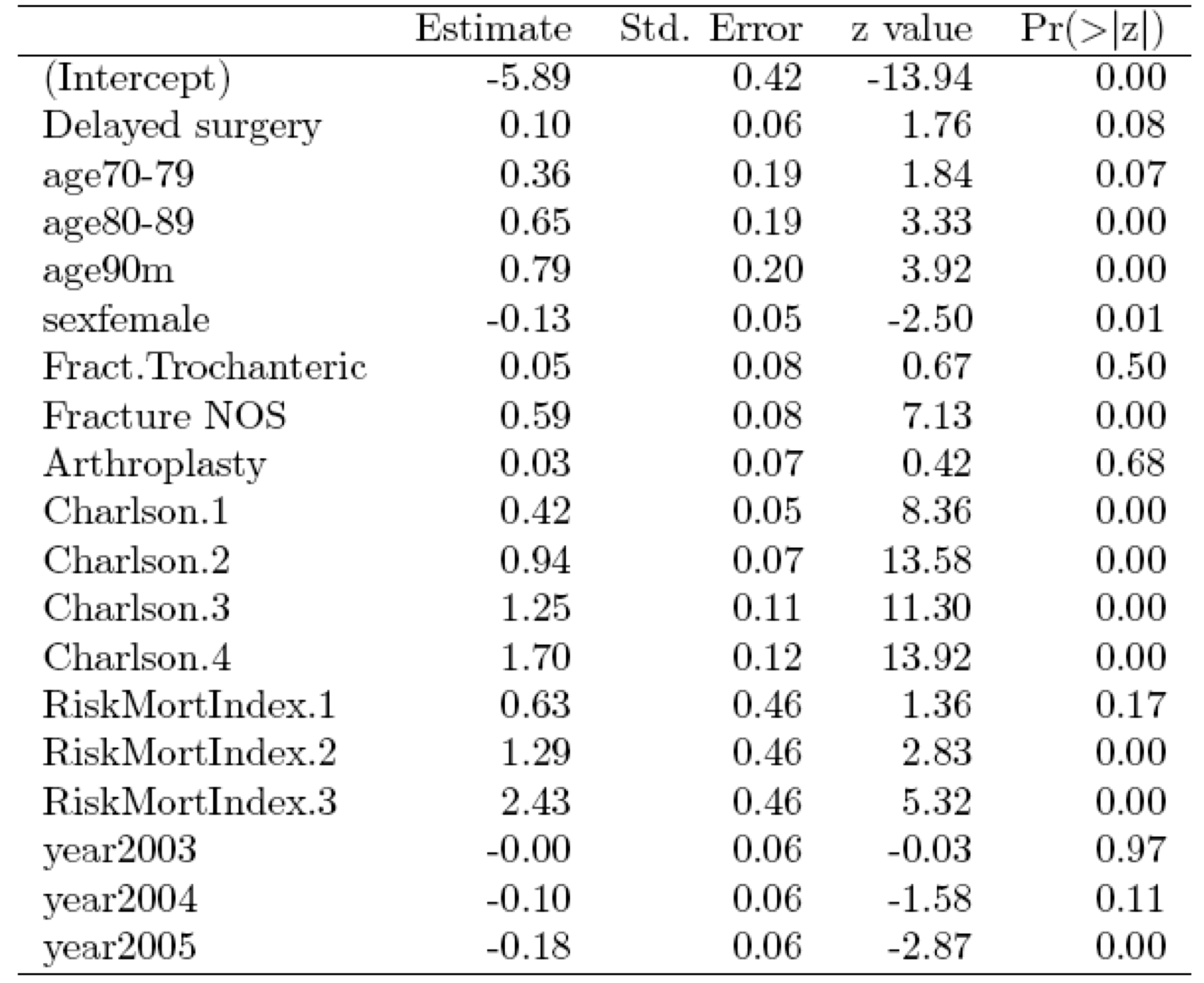** |

| **Additional file 4 - Figure 1. Estimated residuals for all 93 hospitals using the random-intercept (M2M) model.** |
| --- |
| **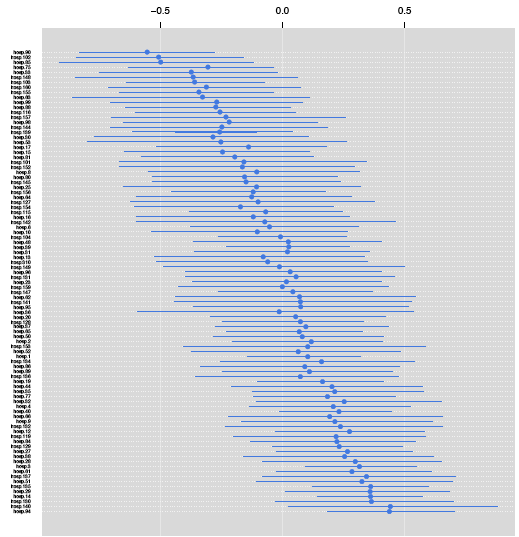** |

**Figure 2** shows the M2L (red) and M2M (black) equivalence. The hospital variance in those models was 0.09364 (M2M) and 0.084504 (M2L)

| **Additional file 4 - Figure 2. Equivalence between M2L (red lines) and M2M (black lines) models.** |
| --- |
| **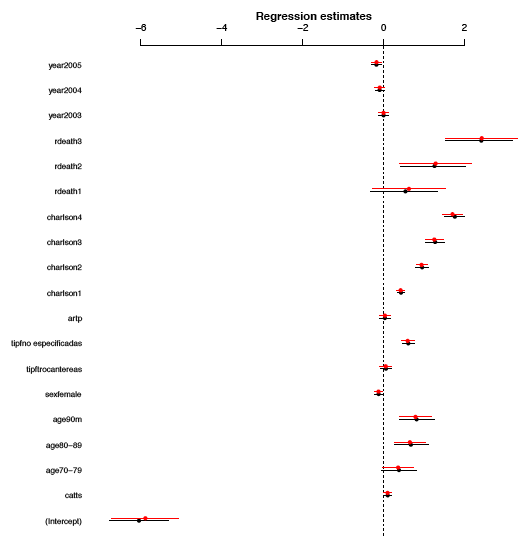** |

Third, a multilevel logistic random coefficient model was used to contrast if the effect of delayed surgery is constant across hospitals (M3L, M3M). This contrast is based on the LR test and the Difference Information Criterion (DIC) between the M3 and M2 models. We also explored some aspects of M3 models like the intercept-random coefficient correlation. **Table 4** shows the M3L model. In this model we incorporated a random-slope on the early surgery variable to allow its effect to vary across hospitals. For this random coefficient, the mean value of the posterior distribution of variance (M3M) was 0.000213, 95%CI: 8.5e-15; 0.0015; and a worse DIC value: 17969.34.

| **Additional file 4 - Table 4. Multilevel logistic random coefficient model (M3L Model).** |
| --- |
| **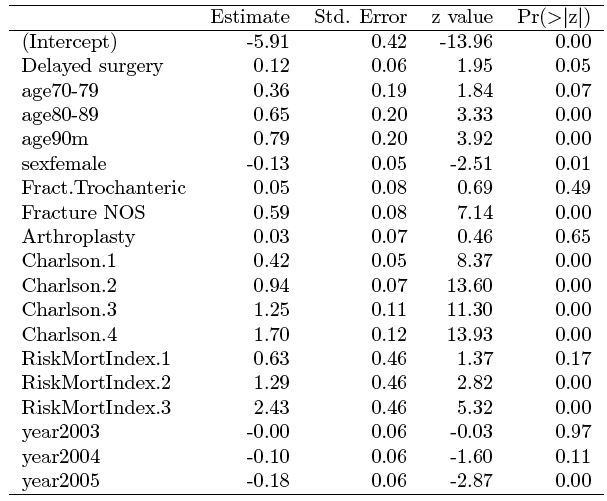** |

**Table 5** shows the contrast between the M3L and M2L models. We use the LR test to analyze whether the delayed surgery effect varies across hospitals. The null hypothesis was that the two additional parameters (coefficient variance and intercept-coefficient covariance) are simultaneously equal to zero. The LR value was 1.15 (p=0.56). Therefore, the null hypothesis was not rejected and we did not find evidence that the delayed surgery effect on mortality would differ across hospitals.

| **Additional file 4 - Table 5. Multilevel logistic random coefficient model (M3L Model).** |
| --- |
| **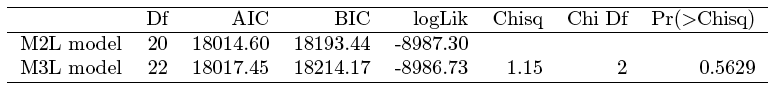** |

**Figure 3** shows the prediction intervals for the bivariate random effects, intercept (on the left) and delayed-surgery random coefficient (on the right). The random effects seem to be best represented by a simple random intercept model, rejecting the hypothesis of heterogeneity effect of delayed surgery across hospitals.

| **Additional file 4 - Figure 3. Prediction intervals for the bivariate random effects intercept (on the left) and delay-surgery random coefficient (on the right).** |
| --- |
| **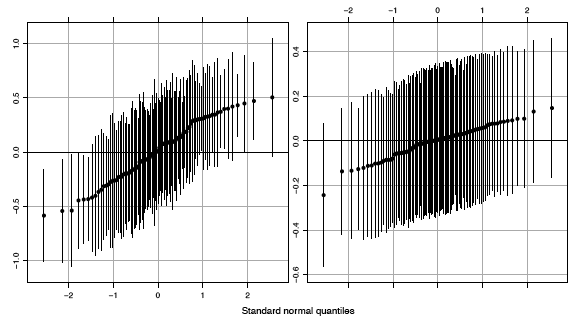** |
