# Supplementary material for: Timing of surgery for hip fracture and in-hospital mortality: a retrospective population-based cohort study in the Spanish National Health System
Source: BMC Health Serv Res. 2012 Jan 18;12:15. doi: 10.1186/1472-6963-12-15 (PMC3292938; doi:10.1186/1472-6963-12-15)
Supplement: Additional file 5 — Instrumental variable approach. Instrumental variable analysis. [file 1472-6963-12-15-S5.DOC]

**Timing of surgery for hip fracture and in-hospital mortality. A retrospective population-based cohort study in the Spanish National Health System.**

**Additional File 5.**

Instrumental variable analysis

Because the severity of early and delayed surgery patients differs, operating delays seem to increase inpatient mortality in the non-adjusted analysis (see Table 3, main article). After adjustment for several patient characteristics, our study shows non-significant mortality differences between both groups (see table 5) but, because we use administrative databases without detailed clinical data, the adjustments were only partial and the bias attributable to the unobserved covariates could affect the study results.

Following a referee’s suggestion, to confirm our results we use the day-of-the-week of admission as an instrumental variable for time to surgery. Because we expect admission day-of-the-week to be related to delayed surgery but not to patient outcomes, we can use this variable to pseudo-randomize patients and test for significant differences between the fraction of patients delayed for surgery and the fraction of patients who died in the hospital by day-of-the-week.

First, we verify that the admission day-of-the-week was related to surgery delay (p<0.001), but not to inhospital mortality (p = 0.98). Second, we estimate the impact of surgery delay in a baseline probit model without the instrumental variable (**Table 1**). Finally, we replicate the previous probit model using the day-of-the-week as an instrumental variable (**Table 2**). The Wald test of exogeneity was 0.06 (p=0.8098), showing that correcting the possible endogeneity of the variable delayed-surgery using the day-of-the-week as an instrumental variable does not alter the study’s conclusions.

| **Additional File 5 - Table 1. Impact of surgery delay on mortality (probit model without the instrumental variable).** | | | | |
| --- | --- | --- | --- | --- |
|  |  | Coeff. | 95CI | p |
| Constant |  | -2.796605 | 3.0853250;-2.5078840 | <0,001 |
| Age | 70-79 years | 0.1620989 | -0.0020649; 0.3262628 | 0.053 |
|  | 80-89 years | 0.2916205 | 0.1254809; 0.4577602 | 0.001 |
|  | 90+ years | 0.3515062 | 0.1788152; 0.5241972 | 0.000 |
| Sex | Woman | -0.0546234 | -0.1031553;-0.0060915 | 0.027 |
| Fracture | Trochanteric | 0.0257886 | -0.0425833; 0.0941605 | 0.460 |
|  | NOS | 0.2545898 | 0.1786959; 0.3304836 | 0.000 |
| Surgery | Arthroplasty | 0.0215882 | -0.0454715; 0.0886479 | 0.528 |
| Charlson | 1 | 0.1863479 | 0.1416478; 0.2310479 | <0,001 |
| index | 2 | 0.4388197 | 0.3724688; 0.5051707 | <0,001 |
|  | 3 | 0.6020446 | 0.4871459; 0.7169432 | <0,001 |
|  | >3 | 0.8745095 | 0.7431954; 1.0058240 | <0,001 |
| Risk | 4-6 | 0.1979286 | -0.1239874; 0.5198446 | 0.228 |
| mortality | 7-12 | 0.4437176 | 0.1218555; 0.7655797 | 0.007 |
| index | >12 | 0.9631949 | 0.6393036; 1.2870860 | <0,001 |
| Year | 2003 | 0.0003030 | -0.0544250; 0.0550310 | 0.991 |
|  | 2004 | -0.0468845 | -0.1023542; 0.0085852 | 0.098 |
|  | 2005 | -0.0817677 | -0.1375018;-0.0260336 | 0.004 |
| Time to surgery | Delayed | 0.0284014 | -0.0180893; 0.0748922 | 0.231 |
| n= 56,482; p<0.0001; r2: 0.0953; Log likelihood = -9029.8809 | | | | |

| **Additional File 5 - Table 2. Impact of surgery delay on mortality (probit model with the instrumental variable).** | | | | |
| --- | --- | --- | --- | --- |
|  |  | Coeff. | 95CI | p |
| Constant |  | -2.8487890 | -3.3582070;-2.3393710 | <0,001 |
| Age | 70-79 years | 0.1631739 | -0.0036160; 0.3299637 | 0.055 |
|  | 80-89 years | 0.2948227 | 0.1229794; 0.4666661 | 0.001 |
|  | 90+ years | 0.3572990 | 0.1668138; 0.5477841 | 0.001 |
| Sex | Woman | -0.0543653 | -0.1053316;-0.0033990 | 0.037 |
| Fracture | Trochanteric | 0.0217552 | -0.0516484; 0.0951588 | 0.561 |
|  | NOS | 0.2520397 | 0.1011180; 0.4029615 | 0.001 |
| Surgery | Arthroplasty | 0.0141784 | -0.0750167; 0.1033734 | 0.755 |
| Charlson | 1 | 0.1831754 | 0.1053467; 0.2610041 | <0,001 |
| index | 2 | 0.4341944 | 0.3347513; 0.5336375 | <0,001 |
|  | 3 | 0.5968146 | 0.4238214; 0.7698077 | <0,001 |
|  | >3 | 0.8678123 | 0.6979165; 1.0377080 | <0,001 |
| Risk | 4-6 | 0.1958007 | -0.1083974; 0.4999989 | 0.207 |
| mortality | 7-12 | 0.4383493 | 0.1208421; 0.7558564 | 0.007 |
| index | >12 | -2.8487890 | 0.6223744; 1.2890120 | <0,001 |
| Year | 2003 | 0.0001213 | -0.0698914; 0.0701340 | 0.997 |
|  | 2004 | -0.0475683 | -0.1106017; 0.0154650 | 0.139 |
|  | 2005 | -0.0842582 | -0.1485341;-0.0199822 | 0.010 |
| Time to surgery | Delayed | 0.1121677 | -0.5986664; 0.8230018 | 0.757 |
|  |  |  |  |  |
|  | /athrho | -0.0360786 | -0.3476956; 0.2755384 | 0.820 |
|  | /lnsigma | -0.8475056 | -0.9111435;-0.7838676 | <0,001 |
|  |  |  |  |  |
|  | rho | -0.0360630 | -0.3343303; 0.2687707 |  |
|  | sigma | 0.4284824 | 0.4020642; 0.4566365 |  |
| n= 56,482; p<0.0001; r2: 0.0953; Log pseudolikelihood = -41305.527. Standard Errors adjusted for 93 hospital clusters.  Instrumented: Time to surgery; Instrumental: day of the week.  Wald test of exogeneity (/athrho = 0): chi2(1) = 0.05; Prob > chi2 = 0.8205 | | | | |
